# Comparison of corneal topography maps of a swept-source OCT biometer and a Scheimpflug device

**DOI:** 10.1007/s10792-026-04088-6

**Published:** 2026-05-06

**Authors:** Michael Müller, Eva Hemkeppler, Myriam Böhm, Tyll Jandewerth, Christoph Lwowski, Thomas Kohnen

**Affiliations:** Department of Ophthalmology, Goethe-University, Theodor-Stern-Kai 7, 60590 Frankfurt am Main, Germany

**Keywords:** Scheimpflug device, Swept-source OCT, Corneal topography maps, Irregular corneas

## Abstract

**Purpose:**

Rating of central corneal topography of a swept-source OCT biometer (IOL Master 700) and tomographic maps from a Scheimpflug device (Pentacam AXL) by different experienced professionals regarding irregularities to support decisions on implanting premium intraocular lenses (IOLs).

**Methods:**

In this prospective study healthy eyes (H), irregular corneas (I) and eyes with previous corneal refractive surgery (P) were randomly selected. Five observers with different experience in corneal map evaluation compared the corneal topography maps of both devices individually. They answered a questionnaire for each picture and eye and the matched pictures. The questionnaire included the similarity in regularity of the cornea, the classification of the pictures regarding the included patient groups and the decision of premium IOL implantation.

**Results:**

The study included 25 eyes per group. The correct category (H, I or P) was reported in 52% to 70% of individual cases. Except one observer (*p* < 0.001) there were no significant difference between the two devices. Most observers rated irregular corneas significantly different than healthy or post refractive surgery eyes (*p* < 0.001). There were significant inter-observer agreements among the three observers for rating implantation premium IOLs 0.137 to 0.374 (*p* < 0.05) and rating the correct category 0.435 (*p* < 0.001).

**Conclusions:**

Irregular corneas were rated as significantly different to healthy or post refractive surgery eyes. Most observers rated the images of the swept-source OCT biometer and the Scheimpflug device equally. Regarding the selection of premium IOL experienced colleagues are preferable.

## Introduction

In the last decades many intraocular lenses, e.g. toric, extended depth of focus (EDOF) or multifocal IOLs (MIOLs), were developed to provide good visual acuity in all distances to cataract patients who want to be spectacle independent after lens exchange [1]. Choosing the best individual IOL for cataract patients is challenging and includes high quality biometry and anatomy measurements of the eyes and additionally the knowledge of the patients’ needs and expectations despite using the best fitting IOL calculation formula [2, 3].A regular shape of the cornea and healthy eye conditions are necessary for premium IOL implantation [4, 5]. To ensure these requirements a good quality biometry and anatomy measurement should be performed. Swept-source OCT is the gold standard in biometry measurements. The IOL Master 700 has an additional new feature which presents the central 4–5 mm anterior and total cornea topography. On the other hand, Scheimpflug technology is the gold standard in anterior segment imaging (corneal tomography using Scheimpflug technique) producing detailed maps of the anterior and total corneal conditions in a diameter of 9 mm. Thus, the shape of the cornea can be interpreted well to decide which IOL fits best for the patient.

The aim of our study was a comparison of the two tomography devices:

- Subjective rating of the separately shown anterior and total corneal maps of the two devices regarding the assessment of the cornea to the group regular (H), irregular (I) and post refractive surgery (P),
- Comparison of the simultaneously presented matched corneal maps of the two devices and
- Comparison of the decision to implant a premium IOL, e.g. toric, EDOF or multifocal IOL based on the anterior and total maps of the biometer or the Scheimpflug device.

Furthermore, we were interested to see if the professional experience of the observers evaluating the corneal maps had an effect on the decision to implant premium IOLs.

## Subjects and methods

### Study design

This prospective study included patients who visited the Department of Ophthalmology, Goethe University, Frankfurt am Main, Germany for anterior segment imaging in the following conditions: cataract surgery, refractive surgery, state of cornea degeneration disease.

### Enrollment of participants

75 patients were enrolled consecutively regarding the inclusion criteria of the study and their medical history. Inclusion criteria were eyes that had good-quality corneal topography maps from a Scheimpflug device and measurements with good quality from the ocular biometer central topography. We consecutively chose 25 eyes for each group: patients with normal corneas (H) (38.32 ± 9.98 years), irregular corneas e.g. due to scarring after a keratitis (I) (41.60 ± 12.58 years), and corneas after refractive surgery like LASIK/PRK (P) (34.96 ± 11.08 years). Randomly, one eye from each subject was included.

The study was conducted from August 2020 to March 2022 and is consistent with the tenets of the Declaration of Helsinki and in compliance with Good Clinical Practice (GCP), including International Harmonization (ICH) Guidelines. The Department of Ophthalmology at Goethe University in Frankfurt am Main received ethical approval prior to enrollment.

### Devices

Anterior and total corneal maps from both a swept-source OCT biometer and a Scheimpflug tomographer were used for this comparison.

#### IOLMaster 700

The IOLMaster 700 uses swept-source-OCT (ss-OCT) technology for the biometry. For the keratometry measurement, the technology is telecentric spot reflection analysis and for the posterior keratometry a combination of spot reflection and OCT. The keratometry uses reflection pictures of 18 spots on the corneal surface in 3 zones and combines the pachymetry to calculate the 3-dimensional corneal maps. Anterior and total axial maps can be displayed in a 4–5 mm central zone depending on the corneal curvature. The corneal axial curvature is presented in a coloured scale in 0.5 D steps. Additionally, there is a quality check which tells the user if the data can be used for analysis.

#### Pentacam AXL

The Pentacam AXL is a Scheimpflug device which presents three-dimensional images of the anterior segment of the eye in a 9 mm diameter. The anterior and the posterior surface of the cornea are calculated in a movable 3D-model. The device additionally measures the biometry of the eye. The quality of the measurement is controlled by a specification which tells the user if the data can be used for interpretation of the anterior segment of the patient’s eye.

### Subjective rating of the corneal maps

The first part of this study was the interpretation of the single corneal maps of the two devices. Five observers with different experience regarding the interpretation of corneal maps were chosen to rate all 300 corneal map pictures (3 different patient groups, 25 eyes per group, 2 corneal maps per eye (anterior and posterior), and 2 devices (Scheimpflug and ss-OCT)). The observers were an experienced cataract, pIOL and corneal refractive surgeon (ES), who uses all kinds of premium IOLs, an experienced cataract surgeon (S), who uses only aspheric and toric IOLs, an experienced ophthalmology resident (ER), a young ophthalmology resident (YR) and an optician experienced in IOL calculation (CO).

The single maps were presented one map at a time anonymously without further information. The observers received instructions to answer the following questions:

1. Is the cornea regular or irregular?
2. To which patient group (healthy, post refractive surgery, irregular cornea) belongs this map?
3. Would you implant an EDOF IOL?
4. Would you implant a toric IOL?
5. Would you implant a multifocal IOL?

The answers were filled in a table by the observers for analysis.

**Fig. 1:**
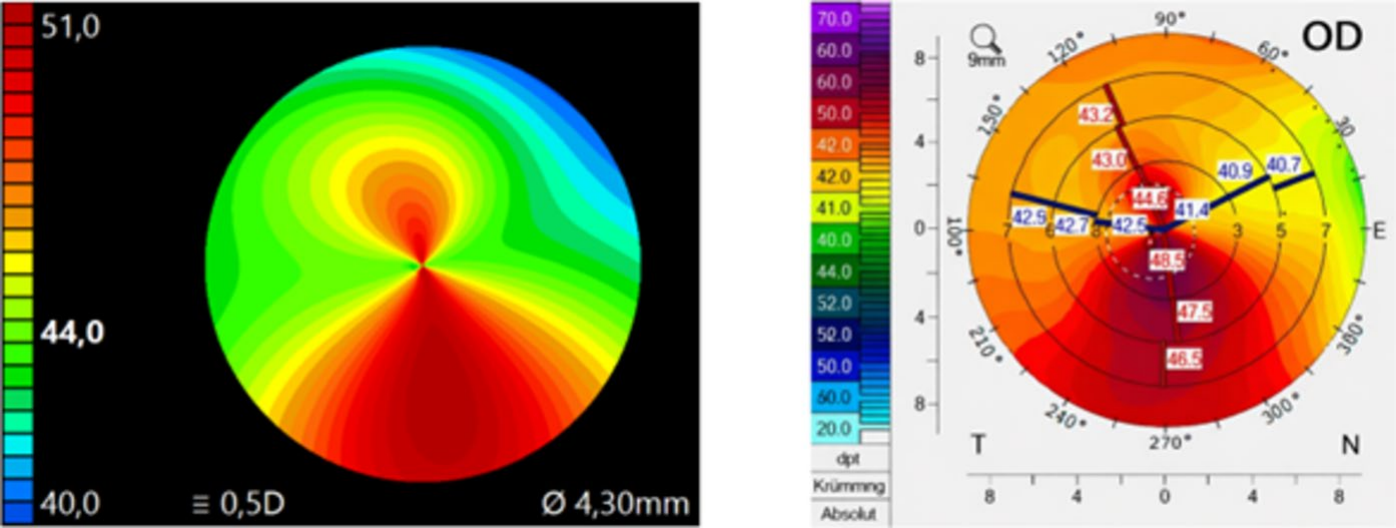
Presentation of the comparatative corneal maps of the same eye of the same patient of the ss-OCT on the left and Scheimpflug device on the right

The second part of the study was the direct comparison of the corneal maps (of the same eye) of the two devices. The pictures of the same eye taken with the different devices were presented simultaneously (Fig. 1). The observers got the instruction to answer the following questions:

1. Are the corneal maps comparable regarding regularity?
2. Are there clinical relevant differences requiring further information to make a decision?
3. Would you implant a toric IOL based on the information of the presented maps of the same eye?
4. Would you implant a multifocal IOL based on the information of the presented maps of the same eye?
5. Would you implant an EDOF IOL based on the information of the presented maps of the same eye?
6. Do you need further examinations to make a decision?

The answers were filled in a table by the observers for analysis.

### Statistical analysis

For the analysis of the subjective rating of the corneal maps an interobserver analysis was conducted. The comparison of the results between these S-OCT and Scheimpflug devices was done by the Chi-Square-Test. The statistical analysis was performed using Microsoft Excel 2016 (Microsoft Corp., USA) and SPSS Statistics (Version 28, IBM Corp., USA). Since this was a pilot exploratory study, a sample size calculation was not feasible.

## Results

A regular cornea enables the decision of implanting premium IOLs, especially EDOF and M-IOLs. In case of irregular corneas the decision of implanting premium IOLs requires fundamental consideration. Thus, the main assignment of corneal topography is to detect normal corneas as normal and to give the opportunity to detect irregularities no matter the aetiology.

At first an inter-observer analysis of all observers was done to measure the agreement of the observers’ answers. Regarding the interpretation guidelines of Landis and Koch, kappa values ≤ 0 as indicating no agreement and 0.01–0.20 as none to slight, 0.21–0.40 as fair, 0.41–0.60 as moderate, 0.61–0.80 as substantial, and 0.81–1.00 as almost perfect agreement The results for all observers show a fair to moderate agreement for the decisions regarding patient category (H, I, and P) (k = 0.435) and regarding toric IOL implantation (k = 0.374) (*p* = 0.05). There is low agreement in EDOF (k = 0.239) and multifocal IOL implantation (k = 0.137) (*p* < 0.05). Due to many outliers while having a closer look on the observers´ answers, a subgroup analysis was done including only the experienced observers (ES, S and ER). The results show better agreement in patient categories (patient category (H, I, and P) (k = 0.500), irregular corneas (k = 0.595) and toric IOL implantation (k = 0.473) (*p* < 0.001). EDOF (k = 0.306) and multifocal IOL implantation (k = 0.188) (*p* < 0.05) was lower. On the basis of these outcomes for further analysis only the data of the experienced observers were used.

The analysis of the evaluation of the single maps for the different observers shows overall a high agreement regarding the assessment of the regularity and the patient group classification. The regularity assessment for the different observers was correct in 84% for ES, 79% for S and 76% for ER. The patient group was correctly identified in 70% for ES, 65% for S and 64% for ER, of the single maps (anterior or total). The agreement between the rating of the observers and the possibility of premium IOL implantation independent of the device shows, that the surgeon who doesn’t implant multifocal IOLs often wouldn’t implant these kind of IOLs either. The inter-observer agreement analysis shows highest agreement between all observers regarding the possible implantation of toric IOLs (ES 58%, S 46%, ER 36%) and the lowest agreement for the possibility of multifocal IOLs (14% vs. up to 27%) (Table 1).

**Table 1:** Results of the single map evaluation of the three experienced observers regarding correct regularity, correct patient group and the possibility to implant premium IOLs

Observer	Regularity correctly detected?	Group correctly detected?	Is an EDOF IOL suitable?	Is a toric IOL suitable?	Is a multifocal IOL suitable?
ES	252 (84%)	210 (70%)	175 (58%)	173 (58%)	82 (27%)
S	237 (79%)	194 (65%)	66 (22%)	138 (46%)	41 (14%)
ER	228 (76%)	192 (64%)	145 (48%)	107 (36%)	49 (16%)

With the exception of S (*p* < 0.01), all observers show no statistically significant difference in rating the correct patient category from ss-OCT biometer or Scheimpflug device (*p* < 0.05) maps. Regarding the corneal regularity, S evaluated the ss-OCT biometer pictures more often correct (88.7%) than the Scheimpflug device pictures (70%). This is may be due to the familiarization of the coloured maps because S is more used to looking at these colour scales in the past. No matter which corneal topography technique was used, at least 3 out of 4 cases were diagnosed correctly regarding the regularity. All observers had higher accordance in correct category in the Scheimpflug device maps than in the ss-OCT maps. Irregular corneas often show the irregularity in the outer diameter of the cornea so the reason for better results in Scheimpflug device measurements might be the larger diameter of the measuring zone (Table 2) [6]. The false positive rate was 28% (ES) over 20% (S) to 12% (ER) in ss-OCT ratings and 14.5% (ES) over 9.5% (S) to 5% (ER) in the Scheimpflug device.

**Table 2:** Results of the single map evaluation of the three experienced observers of IOL Master 700 versus Pentacam AXL corneal maps regarding correct regularity, correct patient group and the possibility of premium IOL implantation

Observer	Regularity correctly detected?		Category correctly detected?		Is EDOF IOL possible?		Is toric IOL possible?		Is multifocal IOL possible?	
ES	110 (73.3%)	114 (76.0%)	64 (42.7%)	110 (73.3%)	81 (54.0%)	94 (62.7%)	95 (63.3%)	78 (52.0%)	43 (28.7%)	39 (26.0%)
S	133 (88.7%)	105 (70.0%)	84 (56.0%)	109 (72.7%)	30 (20.0%)	36 (24.0%)	75 (50.0%)	93 (62.0%)	33 (22.0%)	23 (15.3%)
ER	116 (77.3%)	113 (75.3%)	94 (62.7%)	99 (66.0%)	64 (42.7%)	81 (54.0%)	54 (36.0%)	53 (35.3%)	22 (14.7%)	27 (18.0%)
IOL Master 700/Pentacam AXL										

The results of the comparison of the two devices shows that there is no statistically difference regarding the group classification based on the anterior or total corneal map (*p* < 0.05). All observers rated the regularity of the irregular corneal maps statistically significantly different in the two devices (*p* < 0.05). (Table 3).

**Table 3:** Results of the comparison of the corneal maps evaluation of the three experienced observers of the IOL Master 700 and the Pentacam AXL

Observer	Similar regularity?	Clinically relevant difference?	Similar decision for toric IOL?	Similar decision for multifocal IOL?	Similar decision for EDOF IOL?	Extra measurement necessary?
ES	98 (65%)	85 (43%)	56 (63%)	44 (71%)	99 (66%)	24 (16%)
S	116 (77%)	40 (27%)	105 (70%)	150 (100%)	146 (97%)	17 (11%)
ER	108 (72%)	45 (30%)	117 (78%)	16 (11%)	131 (87%)	7 (5%)

## Discussion

In this study the corneal maps of a ss-OCT biometer and a Scheimpflug device were rated and compared by three observers with different experience in evaluation of corneal maps. The most relevant differences in the presentation of the corneal pictures is the measurement technology (ss-OCT biometer combining telecentric spot reflection analysis vs. Scheimpflug technology) and the diameter of the measurement (ss-OCT biometer 4–5 mm vs. Scheimpflug device 9 mm). In the evaluation of the single maps of both devices the observers had a higher agreement regarding the corneal regularity than for the group classification. In the decision of regularity, the central corneal area (4—5 mm), which is visible in the ss-OCT biometer maps, is most relevant, whereas in the patient group classification (in particular to discriminate I and P) the outer areas of the cornea (> 4 mm) visible in the Scheimpflug device maps become more important [6]. Accordingly, the post-refractive cornea maps were often rated as irregular, which was right, but not correctly categorized to the post-refractive group (P) but rather in the group of irregular eyes (I). In daily practice premium IOLs are sometimes implanted in post-refractive surgery patients but only in rare cases if the cornea irregularity is caused by a corneal disease. In some cases toric IOL implantation is suitable for keratoconus patients but should be considered carefully [7]. Post-refractive surgery eyes must undergo a detailed preoperative examination and an appropriate IOL calculation formula must be used [8–10] to minimize the risk of residual refractive errors and unwanted optical phenomena that may lead to patient unsatisfaction [11, 12]. For this reason, it is necessary, in post-refractive surgery eyes, to obtain a large diameter topography to allow healthcare professionals to make an informed decision on the IOL type. Furthermore, the results of this study show, similar to a previous publication of Kanclerz et al. [13], that the decision of the best fitting IOL for the individual patient varies between observers, even if they are experienced in the implantation of premium IOLs. Therefore, we would recommend, in difficult cases, to have several trained professionals discuss the best IOL option for a given patient.

Another factor which should be considered for a correct interpretation of the corneal maps is the tear film of the patient. The stability of the tear film has a high impact on the quality of the corneal pictures. Corneal pictures of an eye which has a poor tear film often look like irregular corneas even if there is no irregularity in the corneal shape [14].

The comparison of the two devices, which were used in this study, showed good agreement in anterior and total corneal maps in healthy eyes. Wang et al. reported similar results in their paper which compares the corneal maps of the ss-OCT biometer versus a dual Scheimpflug camera system [15]. In corneas which have a history of refractive surgery or irregularity, the maps of the two devices are not completely comparable because of the different diameter of the topographies. Keratoconus eyes, in particular in early-stage, often show characteristic curvatures outside the 4–5 mm zone [16]. In contrast to several studies in which good agreement between corneal topographies generated by the swept-soure OCT and Scheimpflug devices were reported [17–19], this study, where irregular corneas were presented which also influence peripheral parts of the cornea, shows that the outer sections of the corneal maps (larger than 5 mm) can influence the decision regarding regularity of the cornea and possibility of implanting premium IOLs. This study shows that the OCT-based central topography, which presents a central corneal map additionally to the biometry of the eye, works well in normal, healthy eyes. To detect, if an irregularity is due to a previous refractive corneal surgery or a corneal disease, larger diameter topography maps are required to provide enough information for choosing the best suitable IOL for the individual patient. In line with this, Brar et al. who compared the ss-OCT biometer and an older version of the Scheimpflug device, concluded that due to the snaller zone of the ss-OCT topography off centered irregularities are not predictable [20]. So in these cases an additional Scheimpflug examination is necessary for a premium IOL decision.

Although a good measurement quality of the corneal maps was considered, the limitation of the study is that the tear film was not evaluated and may lead to irregularity of the corneal maps.

In conclusion, it is not surprising, that the quality of detecting corneal regularities and the right diagnosis of patient groups is dependent on the physician’s experience. The new topographic feature of ss-OCT biometer can be used for screening if a healthy eye is present, where premium IOLs might be a good option. In the differentiation of irregular corneas (between corneal pathology or post corneal refractive surgery) a measuring zone larger than 4–5 mm is preferable to be able to make a correct classification.

## Data Availability

No datasets were generated or analysed during the current study.
